# Comparative analysis of ventricular stiffness across species

**DOI:** 10.14814/phy2.16013

**Published:** 2024-04-21

**Authors:** Yuu Usui, Akira Hanashima, Ken Hashimoto, Misaki Kimoto, Momoko Ohira, Satoshi Mohri

**Affiliations:** ^1^ First Department of Physiology Kawasaki Medical School Kurashiki Okayama Japan

**Keywords:** collagen, end‐diastolic pressure‐volume relationship, Titin (Connectin), ventricular stiffness

## Abstract

Investigating ventricular diastolic properties is crucial for understanding the physiological cardiac functions in organisms and unraveling the pathological mechanisms of cardiovascular disorders. Ventricular stiffness, a fundamental parameter that defines ventricular diastolic functions in chordates, is typically analyzed using the end‐diastolic pressure–volume relationship (EDPVR). However, comparing ventricular stiffness accurately across chambers of varying maximum volume capacities has been a long‐standing challenge. As one of the solutions to this problem, we propose calculating a relative ventricular stiffness index by applying an exponential approximation formula to the EDPVR plot data of the relationship between ventricular pressure and values of normalized ventricular volume by the ventricular weight. This article reviews the potential, utility, and limitations of using normalized EDPVR analysis in recent studies. Herein, we measured and ranked ventricular stiffness in differently sized and shaped chambers using ex vivo ventricular pressure‐volume analysis data from four animals: Wistar rats, red‐eared slider turtles, masu salmon, and cherry salmon. Furthermore, we have discussed the mechanical effects of intracellular and extracellular viscoelastic components, Titin (Connectin) filaments, collagens, physiological sarcomere length, and other factors that govern ventricular stiffness. Our review provides insights into the comparison of ventricular stiffness in different‐sized ventricles between heterologous and homologous species, including non‐model organisms.

## Graphical abstract

This review highlights recent studies assessing ventricular stiffness in differently sized heart chambers using end‐diastolic pressure–volume relationship data from three chordates: Wistar rats, red‐eared slider turtles, and masu or cherry salmon. The relative ventricular stiffness indices, normalizing ventricular volume to the ventricular weight, may be useful for comparing ventricular diastolic properties among heterologous and homologous species, including non‐model organisms. In addition, the review delves into the several mechanical effects of the intracellular and extracellular viscoelastic components regulating ventricular stiffness.

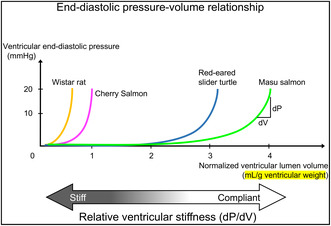

## 1 INTRODUCTION

Accurately evaluating ventricular diastolic properties is crucial for interpreting cardiac functions as a blood pump and understanding the pathological mechanisms of cardiovascular disorders. Investigating ventricular diastolic properties using pressure–volume analysis in the ventricle has been a cornerstone of cardiac dynamics studies (Frank, 1895; Patterson & Starling, 1914). Notably, several studies have aimed to provide comprehensive insights into cardiac functions (Burkhoff et al., 2005; Mirsky, 1984; Suga, 1969). Currently, ventricular diastolic functions have been examined in various species, ranging from mammals to fish, utilizing cardiac stiffness analyses, cardiac hemodynamics imaging, and molecular biological experiments, demonstrating that ventricular diastolic functions differ among chordates (Burkhoff et al., 2005; Farrell, 1991; Kraner & Ogden, 1956; Warburton & Fritsche, 2000; Wu et al., 2004). For instance, in healthy adult humans, early diastolic ventricular filling driven by releasing the elastic energy stored during deformation in the ventricular systole dominates atrioventricular inflows, whereas ventricular filling in many fishes primarily depends on atrial contraction.

A counterclockwise loop emerged with each cardiac cycle when sequential pressure and volume dynamics of the mammalian left ventricle were recorded and plotted on a graph, with ventricular pressure on the vertical axis and ventricular lumen volume on the horizontal axis (Burkhoff et al., 2003). Based on hemodynamic principles, the physiological significance of the ventricular pressure‐volume relationship was confirmed by deterministic relationships in myocardial energetic studies, such as oxygen consumption and time‐varying elastic models (Suga, 1979; Suga, 1990). Moreover, analysis of ventricular pressure–volume dynamic patterns has been demonstrated to be effective in inferring ventricular systolic and diastolic functions, aiding in understanding cardiac physiology and pathology (Burkhoff et al., 2003).

**FIGURE 1:**
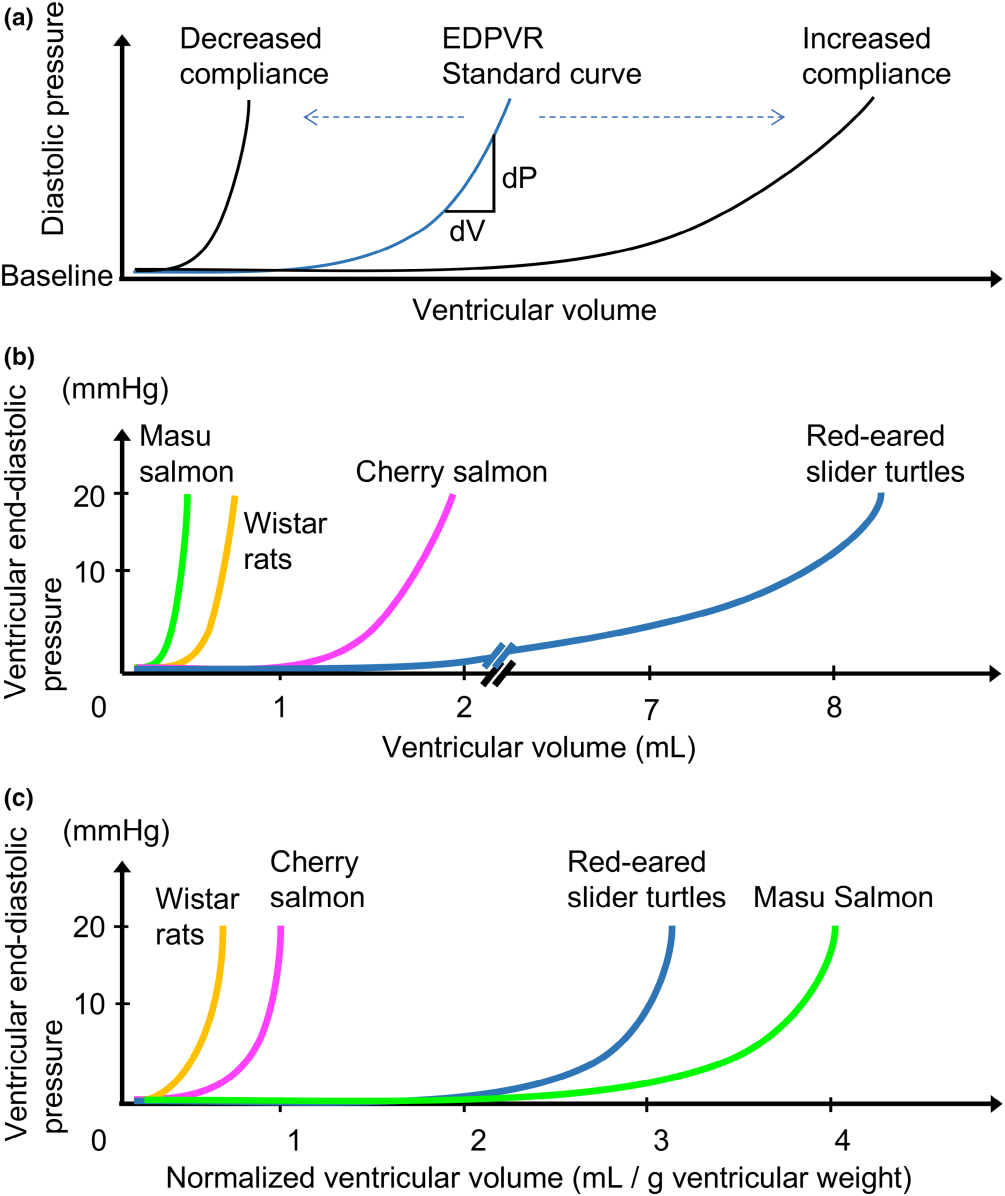
End‐diastolic pressure–volume relationship (EDPVR) curves. (a) An image of EDPVR curves in the different scores of ventricular stiffness. The horizontal axis represents ventricular volume, and the vertical axis indicates ventricular pressure. The left, middle, and right curves indicate that the chamber material is stiff, standard, and compliant, respectively. (b) EDPVR curves for (left) ventricles of Wistar rats (yellow), red‐eared slider turtles (blue), masu salmon (light green), and cherry salmon (magenta). (c) EDPVR plots in which the previous curves (B) were normalized by their corresponding ventricular weights. The horizontal axis represents ventricular volume normalized by ventricular weight.

At the outset, it is important to appreciate that there are two distinct, although intimately interrelated, aspects to assessing cardiac stiffness (Burkhoff et al., 2005; Villalobos Lizardi et al., 2022). One aspect involves the assessment of ventricular stiffness, which pertains to the systolic and diastolic properties of the ventricle as a hemodynamic pump. The other aspect focuses on myocardial stiffness, which assesses the intrinsic properties of the myocardium and cardiomyocyte. Ventricular stiffness, or its reciprocal, ventricular compliance, is an important parameter for defining ventricular filling and has been examined using the ventricular pressure–volume analysis (Burkhoff et al., 2005). The change in ventricular end‐diastolic pressure relative to the change in ventricular volume (*dP*/*dV*) during the ventricular filling phase, from mitral valve opening to closing, represents ventricular stiffness and demonstrates a nonlinear curve (Figure 1a) (Diamond et al., 1971; Grossman et al., 1973). These end‐diastolic pressure–volume relationship (EDPVR) curves deform their slopes to reflect ventricular material properties (sarcomere extensibility and extracellular matrix [ECM] accumulation), physiological remodeling with normal growth, pathological remodeling (fibrosis, ischemia, edema, cardiac hypertrophy, heart failure, and myocardial infarction), and structural changes (malformation of the trabeculae carnies and valves) (Mirsky & Pasipoularides, 1990; Wisneski & Bristow, 1978). Therefore, ventricular stiffness reflects myocardial properties, ventricular structure, and ventricular geometry. Evaluating EDPVR curves is clinically significant, and the curve's position changes depending on the types of ventricular impairment. For instance, the curve shifts to the left in heart failure with preserved ejection fraction (HFpEF) and to the right in heart failure with reduced ejection fraction (HFrEF), compared with that in a healthy heart (Borlaug, 2014; Schwarzl et al., 2016). However, EDPVR analysis using conductance catheters has limited clinical applicability because of its high invasiveness. Recently, minimally invasive measurement methods have been proposed to predict the ventricular stiffness index from a single heartbeat and echography imaging (Kasner et al., 2015; Klotz et al., 2006).

Consequently, assuming that two differently sized hearts have geometric similarities associated with development of the thickness of the ventricular walls and exhibit equal myocardial stiffness, the larger ventricle should exhibit lower ventricular pressure than the smaller one when both are filled with the same blood volume. In this case, ventricular stiffness depends on ventricular lumen volume, and the two hearts exhibit different *dP*/*dV*. Therefore, comparing ventricular stiffness in chambers with varying volume capacities poses a challenge (Burkhoff et al., 2003; Burkhoff et al., 2005). To solve this problem, approaches are needed to calculate the ventricular stiffness index from the normalized EDPVR curve, considering each sample's ventricular lumen volume and mass. Recently, we compared ventricular stiffness among other species using the normalized EDPVR assessment system and evaluated the relative diastolic ventricular stiffness of four types of hearts from three species: Wistar rats (*Rattus norvegicus*), red‐eared slider turtles (*Trachemys scripta elegans*), masu salmon (landlocked type, *Oncorhynchus masou masou*), and cherry salmon (sea‐run type) (Honda et al., 2018; Usui et al., 2022). These animals were selected because they met the experimental requirements of availability, ease of husbandry, and ventricular size, allowing the stable placement of conductance catheters.

In this review, we consolidate our findings with literature evaluating ventricular stiffness in chambers of different sizes across species. In addition, we discuss the mechanical effects of intracellular and extracellular viscoelastic components that govern ventricular stiffness.

## 2 RELATIVE ANALYSIS OF VENTRICULAR STIFFNESS

This section highlights the potential challenges in comparing the ventricular stiffness in ventricles of different sizes and proposes solutions. Initially, we assumed three types of ventricles with different stiffness levels (standard, stiff, and compliant), all of the same size. Next, we plotted their end‐diastolic pressure–volume data (Figure 1a). Herein, the middle curve was assumed to be pressure–volume plots of a healthy ventricle as a standard EDPVR. The stiffer ventricle exhibited a leftward shift and a steeper EDPVR curve than the standard (Maurer et al., 2006). Conversely, the ventricle with increased compliance displayed a gentle slope and a rightward shift in the EDPVR curve (Maurer et al., 2006).

Each EDPVR curve was described as an exponential fit based on the following equation:

(1) $$P={\mathrm{\alpha e}}^{\beta \;V}+\gamma ,$$

where *P* indicates the ventricular pressure and *α*, *β*, and *γ* are constants describing the ventricular exponential pressure–volume property (Burkhoff et al., 2005; Mirsky, 1976). The constant *β* is the ventricular stiffness index representing the curve's slope. *γ* is the intercept on the pressure axis (*α* = −*γ*), and the curve‐fitting formula was forced through the origin. *V* represents the ventricular volume, synonymous with the volume of injected saline in a ventricle to measure ventricular pressure. In EDPVR analysis, saline was infused at a constant flow rate using an auto‐infusion pump into the ventricles, ligated in the proximal ventricular side of the aorta or bulbous arteriosus to prevent leakage. In addition to Equation 1, several curve‐fitting formulas have been proposed to calculate the ventricular stiffness index (Burkhoff et al., 2005). In Equation 1, *β* is expressed in milliliters^−1^ and depends on the ventricular size (Mirsky, 1976). Therefore, as a next step, if the three ventricles had different sizes, the position relationships of the EDPVR curves may not correlate with the assumed ventricular stiffness. A previous study proposed a strategy that compared the dimensionless ventricular stiffness index *β*, multiplied by end‐diastolic volume normalized in the volume dimension from 0 to 30 mmHg (Klotz et al., 2006). Volume‐normalized EDPVR curves showed a similar shape independent of the heart‐healthy conditions, and the uniqueness of this curve contributed to estimating EDPVR (Klotz et al., 2006). Normalizing EDPVR using optimized parameters has been suggested to allow for finding the curve's uniqueness. Clinical investigations have sometimes normalized ventricular volume by body surface area (Gaasch et al., 1976). The relation between ventricular stiffness and ventricular volume/myocardial volume ratio has been found to distinguish between ventricular hypertrophy and increasing ventricular stiffness (Gaasch et al., 1976). The stress (force per unit myocardial area)‐strain (segment length relative to reference length) relationship analysis is a typical evaluation method for assessing intrinsic myocardial stiffness, locally applied to a small volume of the myocardium, independent of ventricular size (Takaoka et al., 2002). It is based on Laplace's law and assumes an ellipsoidal spherical ventricle (Mirsky et al., 1987). Rat ventricles are mirrored as ellipses. The red‐eared slider turtle ventricles also exhibit an ellipsoidal shape when the gubernaculum cordis is detached (Honda et al., 2018). However, their ventricles exhibit a cone shape when connected to the gubernaculum cordis. Masu and cherry salmons have pyramidal‐shaped ventricles (Usui et al., 2022). Therefore, the stress–strain relationship analysis is not necessarily appropriate for measuring myocardial stiffness across animal species or would require complicated calculations to compensate for their ventricular morphology to compare the hearts of different structures. The ventricular systolic index, *E*_max_, was strongly influenced by ventricular size. This problem was solved by converting the systolic pressure–volume relationship into an end‐systolic force–length relationship per unit myocardial mass of the ventricular wall (Suga et al., 1984). The ventricular lumens of red‐eared slider turtles and masu and cherry salmons are arranged in trabeculated myocardial cells. Considering their spongy layer, correction for diastolic ventricular stiffness is difficult in this calculation method. Therefore, appropriately corrected ventricular stiffness indices must be explored to compare ventricular diastolic functions across a wide range of animal species and ventricles of different sizes by modifying the curve‐fitting formulas of EDPVRs.

To overcome this challenge, we calculated the ventricle stiffness using EDPVR curves based on the relationship between ventricular lumen volume per gram of ventricle and ventricular pressure. We substituted the following formula into parameter *V* in Equation 1:

(2) $$V=v_{t}m^{-1},$$

where *V* is the total volume of saline infused into the ventricle at time *t*, *v*_0_ = 0, and it is described in milliliters; m represents the ventricular weight. In this case, *β’* has the units of gram × milliliters^−1^, and the ventricle size is considered.

Our research group previously conducted ex vivo EDPVR analysis on the hearts of Wistar rats, red‐eared slider turtles, and masu and cherry salmons using the same experimental system (Table 1) (Honda et al., 2018; Usui et al., 2022). Saline was injected at a constant rate into their (left) ventricles. Their pressure‐volume relationships were plotted on the same graph until the intraventricular pressure reached ~ 20 mmHg (Figure 1b). Subsequently, EDPVR curves were replotted for the ventricles of Wistar rats, red‐eared slider turtles, and masu and cherry salmons, with ventricular volume divided by ventricular weight as the horizontal axis (Figure 1c). The relative ventricular stiffness indices from the replotted normalized EDPVR curves were calculated using Equation 1 (Table 1). Consequently, the relative ventricular stiffness indices were rearranged in the order of Wistar rats > cherry salmon > red‐eared slider turtles > masu salmon. The Wistar rat's ventricle was estimated to be the stiffest of the four types. These relative ventricular stiffness indices have also been calculated for three frog species, namely aquatic African clawed frogs (*Xenopus laevis*), terrestrial or semiaquatic black‐spotted pond frogs (*Pelophylax nigromaculatus*), and Japanese common toads (*Bufo japonicus formosus*), by the same normalization method (Ito et al., 2023). The ventricles of black‐spotted pond frogs and Japanese common toads are shown to be stiffer than those of African clawed frogs, implying that their ventricles undergo stiffening in the process of adapting to terrestrial life (Ito et al., 2021). The normalized EDPVR curves may help evaluate the relative ventricular stiffness among various animals and provide a comprehensive understanding of their ventricular diastolic properties.

**TABLE 1:** Ventricular stiffness indices.

Animal	Wistar rat	Red‐eared slider turtle	Masu salmon	Cherry salmon
*N*	4	4	4	3
*β*: Ventricular stiffness index	93.0 ± 22.7	1.02 ± 0.22	12.8 ± 2.42	1.64 ± 0.93
*β’*: Ventricular stiffness index normalized by ventricular weight	99.0 ± 7.3	2.07 ± 0.62	1.04 ± 0.19	2.21 ± 0.74
Ventricular weight (g)	0.73 ± 0.04	2.0 ± 0.36	0.08 ± 0.01	1.48 ± 0.36
References	Honda et al. (2018)	Honda et al. (2018)	Usui et al. (2022)	Usui et al. (2022)

*Note*: Values are presented as the mean ± standard deviations.

Protocol of ex vivo end‐diastolic pressure–volume relationship (EDPVR) analysis: Experimental animals were euthanized, and their hearts were removed. A catheter was inserted into the fresh ventricle through the atrioventricular valve and fixed by ligation. The ventricular lumen was washed out using phosphate‐buffered saline with 10 U/mL heparin and 10 mM 2,3‐butanedione monoxime. The aortic valve was then ligated. To measure the ventricular pressure, saline solution was injected into the ventricles using an infusion pump (TE‐311, Terumo Corp.). The ventricular pressure data were recorded at 400 Hz using an analysis software (LabChart 7, ADInstruments Pty Ltd.) by mediating a transducer (DT‐XX, Argon Medical Devices, Inc.), an amplifier (AP‐641G, NIHON KOHDEN Corp.), and an AD converter (Power Lab 8/30, ADInstruments Pty Ltd.). Calibration of the pressure in the measurement equipment was performed for each trial. The sensor was connected to the sphygmomanometer before and after pressure measurement. The sphygmomanometer (Sanden Medical Industry Corp.) was operated in the following order: 0, 50, 0 mmHg. Next, the ventricular lumen fluid was drained, and ventricular weights were measured using an electronic balance. The ventricular stiffness index *β* was calculated from the EDPVR plots in Figure 1b. In these plots, the x‐axis represented the ventricular volume, equivalent to the volume of saline solution injected by the infusion pump, while the *y*‐axis represented the diastolic ventricular pressure. The ventricular stiffness index *β*’, normalized by ventricular weight, was calculated from plots in Figure 1c where the *x*‐axis represented the ventricular volume per gram of ventricle—computed by dividing the ventricular volume by ventricular weight—and the *y*‐axis represented the ventricular pressure. The curve‐fitting equation of EDPVR plots described the exponential growth with a constant doubling time. For detailed information on curve fitting, refer to the RELATIVE ANALYSIS OF VENTRICULAR STIFFNESS in the main text.

## 3 ELASTIC COMPONENTS OF THE VENTRICULAR MYOCARDIUM

This section discusses factors contributing to inter‐ and intra‐species differences in ventricular stiffness, supported by relevant data. We believe that the difference in ventricular stiffness among Wistar rats, red‐eared slider turtles, and two salmon fishes could be explained by factors, such as the number of amino acids in the spring functional region of Titin (Connectin, encoded by *ttn*) expressed in the ventricles, collagen fiber deposits in the ventricles, and the range differences in physiological sarcomere length in the cardiomyocyte.

### 3.1 Elastic region length of titins

**FIGURE 2:**
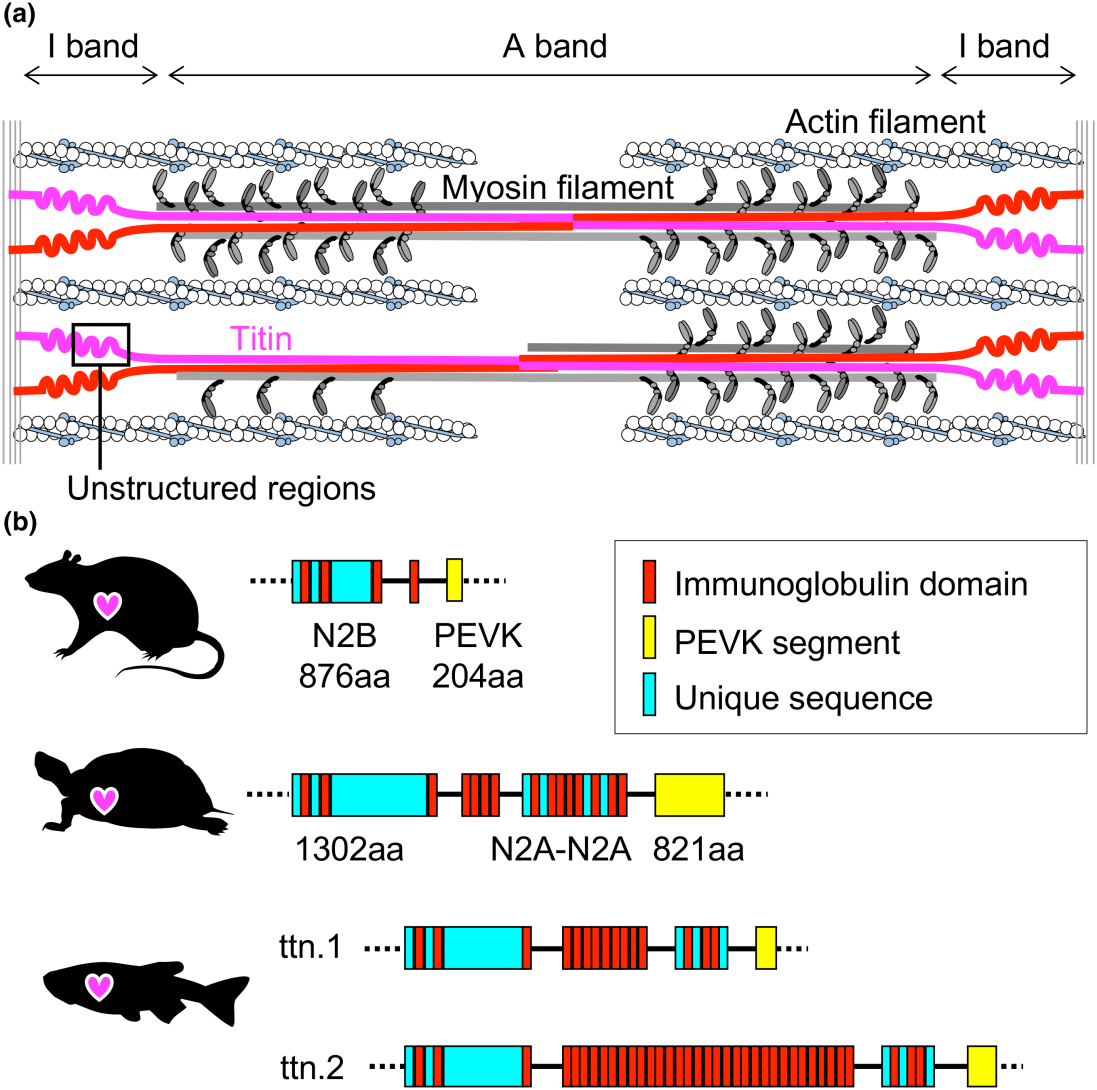
Comparison of unstructured regions in Titins in ventricles. (a) An image of sarcomere structures in a cardiomyocyte. Titin filaments are shown in magenta or red. A box with a black line indicates an unstructured region, an elastic function domain in Titin. Actin filaments and troponins are indicated in white and blue, respectively. Myosin filaments are colored light and dark gray. (b) Domain components in the unstructured region of Titins mainly expressed in the ventricles of Wistar rats (Accession number: LC269844), red‐eared slider turtles (LC269845), and zebrafish (ttn.1: LC168152 And ttn.2: LC168151). Red, yellow, and pale blue boxes indicate the immunoglobulin domain, PEVK segment, and unique sequence, respectively.

Sarcomeres in cardiomyocytes mainly comprise three filaments, namely actin, myosin, and Titin (Figure 2a). Titins are high‐molecular‐weight proteins (3–4 MDa) that extend across half the sarcomere length from the *Z*‐disc to the *M*‐line (Maruyama et al., 1976; Wang et al., 1979). Titin is responsible for generating passive tension in cardiomyocytes (Granzier & Irving, 1995; Linke et al., 1994). In a sarcomere's I‐band, Titins have an unstructured region that reserves elastic potential energy, the N2A element, the N2B element, the middle tandem immunoglobulin‐like (Ig) segment, and the PEVK (proline‐glutamate‐valine‐lysine resides repeats and E‐rich motifs) segment (Bang et al., 2001; Freiburg et al., 2000). Conformational changes in these elastic elements are believed to support sarcomere function as biological springs. Human cardiomyocytes express several Titin isoforms, including N2B, N2BA, Novex1, and Novex2, and two short isoforms, Novex3 (0.63 MDa) and Cronos (2.2 MDa) (Bang et al., 2001; Freiburg et al., 2000). The N2BA isoform contains the N2B and N2A elements. The N2B isoform lacks the N2A element and the middle tandem Ig segment, except for I27, by splicing. It has fewer amino acids in its PEVK segment than the N2BA isoform. Novex1 has the N2B element, I16, I27, and PEVK segment in the unstructured region; Novex2 has the I17, I27, and PEVK segment in the unstructured region; and Novex3 has a unique C‐terminal sequence, localizes in the nucleus during the embryonic stage of mice, and regulates nuclear stiffness in cardiomyocytes (Hashimoto et al., 2018). Cronos lacks the N‐terminal region of Titin and supports partial sarcomere formation. The expression patterns of Titin isoforms in the heart have been extensively studied in mammals (mice, rats, rabbits, dogs, humans, pigs, cows, and sheep) (Cazorla et al., 2000; Locker & Wild, 1986), birds (chickens and thrushes) (Locker & Wild, 1986), and fish (masu salmon, cherry salmon, zebrafish (*Danio rerio*), and rainbow trout (*Oncorhynchus mykiss*)) (Hanashima et al., 2017; Patrick et al., 2010; Usui et al., 2022). Titin isoforms, including N2B elements, are found in cardiomyocytes but not skeletal muscle. The ratio of N2B to N2BA isoforms is linked to the ventricular diastolic function. The myocardium of neonates shows a higher expression ratio of the N2BA isoforms and is more compliant than that of the adult heart (Lahmers et al., 2004). Hearts of patients with diastolic left ventricular dysfunction, characterized by increasing passive tension, as well as athletes' hearts show a higher expression ratio of the N2BA isoform relative to healthy hearts (Hidalgo & Granzier, 2013; Kellermayer et al., 2021; Trombitas et al., 2001). Sprague Dawley rats with *rbm20* knockout display diastolic dysfunction due to impaired splicing out of the N2A element and increased expression of the N2BA isoform (Guo et al., 2012). Mutations in *TTN* and *RBM20* account for approximately 25% and 3% of cases of dilated cardiomyopathy in humans, respectively (Guo et al., 2012; Herman et al., 2012). Consequently, regulating cardiac stiffness through the expression patterns of Titins with longer or shorter unstructured regions has emerged as a novel therapeutic approach for cardiomyopathy. Mammals have only one *ttn* gene in their genome. In contrast, two *ttn* genes, *ttn.1* and *ttn.2*, have been identified in teleosts, such as brown trout (*Salmo trutta*, GeneID: 115155650 and 115155651) and zebrafish (GeneID: 100001684 and 317731; Figure 2b). Notably, multiple isoforms of *ttn.1* and *ttn.2* are expressed in the zebrafish heart (Hanashima et al., 2017). The diversity of *ttn* genes and Titin isoforms might also be implicated in the regulation of myocardial passive stiffness. Furthermore, post‐translated modifications (oxidation, phosphorylation, and deacetylation) in the N2B element, middle tandem Ig segment, and PEVK segment regulate the stiffness of the ventricular myocardium (Koser et al., 2019).

A major Titin expressed in the left ventricles of Wistar rats has a lower molecular weight than that expressed in the red‐eared slider turtle ventricles (Wistar rats: 3.0 MDa, red‐eared slider turtles: 3.5 MDa) (Honda et al., 2018). The left ventricles of Wistar rats predominantly expressed the N2B isoform (Figure 2b). However, the red‐eared slider turtle ventricles expressed the N2BA isoform, containing both N2B and tandem N2A elements. The N2B element includes 876 and 1302 amino acids in Wistar rats and red‐eared slider turtles, respectively. The PEVK segment comprises 204 and 821 amino acids in Wistar rats and red‐eared slider turtles, respectively. Based on the comparison of these, it has been predicted that the ventricular myocardium in red‐eared slider turtles is more compliant than that in Wistar rats (Honda et al., 2018). This prediction is consistent with normalized EDPVR analysis results, which demonstrate that Wistar rat's ventricles are stiffer than red‐eared slider turtle ventricles (Figure 1c and Table 1). Therefore, the length of the unstructured region and the number of amino acids constituting Titin's N2B element and PEVK segment determine the magnitude of passive tension generation in cardiomyocytes. By extension, obtaining the amino acid sequence of Titin expressed in the hearts of experimental animals under study from the genomes or gene expression platforms may provide a rough prediction of their ventricular diastolic properties. The hearts of red‐eared slider turtles and fishes express longer unstructured regions of Titins than those of mammals, which may be used as a model for dilated cardiomyopathy without gene manipulations.

The viscoelastic properties of cardiomyocytes are not solely dependent on Titin. Recently, it was reported that stabilizing the non‐sarcomeric cytoskeleton contributes to cardiac stiffness. Emerging evidence suggests that posttranslational modifications (acetylation and detyrosination) and polymerization of microtubules alter cardiomyocyte viscoelastic stiffness, and these changes are associated with pathological cardiac remodeling through tubulin stabilization (Caporizzo et al., 2019). Tubulin stabilization and metabolization are therapeutic targets for reducing stiffness in heart failure (Caporizzo & Prosser, 2022). The contribution of passive tension generated by microtubules and intermediate filaments to ventricular stiffness has been investigated alongside Titin and collagen. However, a report suggests that their contribution is smaller than that of Titin and collagen in healthy Sprague Dawley rat myocardium, particularly within the range of the mammalian physiological sarcomere length (Granzier & Irving, 1995).

### 3.2 Collagen fiber deposition in the ventricular myocardium

The ECMs in the animal heart support the tissue structure, being localized between myocardial cells and around blood vessels (Eghbali & Weber, 1990). The cardiac ECMs form a complex network within the inter‐myocardial cell space, consisting of various structural and non‐structural proteins, including glycoproteins (several collagens, fibronectin, laminin, thrombospondins, secreted protein acidic and rich in cysteine, tenascin, osteopontin, periostin, and CCNs), elastin, glycosaminoglycans (hyaluronan and heparan sulfate), and proteoglycans (versican, neurocan, brevican, aggrecan, perlecan, type XVIII collagen, agrin, syndecans, and biglycan) (Rienks et al., 2014). In response to aging, pressure overload, dilated myopathy, HFpEF, and heart inflammation, cardiac ECMs are secreted or degraded in the heart to maintain homeostasis of the cardiovascular system (Bashey et al., 1992; Nikolov & Popovski, 2022). The hearts of neonatal mammals, urodeles, and teleosts contain abundant cardiac ECMs, such as fibronectin and periostin, with a low percentage of collagens. In contrast, the hearts of adult mammals exhibit reduced levels of fibronectin, becoming stiff with a high percentage of collagens (Hortells et al., 2019).

Collagens are the main fibrous component of the cardiac ECMs (Bashey et al., 1992; Nikolov & Popovski, 2022). In patients with heart failure, collagen accumulation is associated with the severity of ventricular diastolic dysfunction (Mirsky & Pasipoularides, 1980; Peterson et al., 1978). In particular, myocardial fibrosis, characterized by the excess deposition of ECMs, including abnormal collagen architectures, is observed in varying degrees in patients with HFpEF and is associated with a worse prognosis, as supported by a study using late gadolinium‐enhanced magnetic resonance imaging and autopsy (Shah et al., 2020). In addition, a large experimental animal model study shows that the progression of HFpEF from pressure overload is accompanied by pathologic shifts in the collagen matrix microstructure, in addition to fibrosis induced by the collagen content (Torres et al., 2020). These results underscore the relevance of collagen in myocardial fibrosis. The collagen superfamily includes 28 genes in humans, and several valuations exist by modification of α‐chains and isoforms (Ricard‐Blum, 2011). While there are species‐specific differences in collagen types among mammals, previous reports have identified 24 types of collagens expressed in the heart, including those implicated in heart disease (Frangogiannis, 2019). The epimysium and perimysium in adult mammals primarily contain type I collagen (Heeneman et al., 2003). Type I collagen constitutes approximately 85% of cardiac ECMs. Type III collagen is expressed in the endomysium, while types V and XI collagens participate in valve formation. Types XIII, XVII, XXI, XXII, and XXV collagens are minimally expressed in the myocardium. Type XXVII collagen is involved in coronary vessel formation, type VI collagen is expressed in myocardial vessels, and type IV collagen is found in the basement membrane (Esmaeili et al., 2020; Frangogiannis, 2019; Heeneman et al., 2003; Nikolov & Popovski, 2022). Zebrafish possess 58 collagen genes, and the expression of 36 collagen genes has been identified in their hearts (Nauroy et al., 2018; Sarohi et al., 2022). Zebrafish predominantly express type I collagens (*col1a1a*, *col1a1b*, and *col1a2*), followed by types V, VI, and IV collagens (Sarohi et al., 2022). The cardiac ECMs of adult mammals and urodeles involve type III collagen; however, there is no expression of type III collagen in the cardiac ECMs of teleosts (Hortells et al., 2019).

In the ventricular myocardium, collagen is cross‐linked through the catalysis of lysyl oxidases, transglutaminases, and non‐enzymatic advanced glycation end products and accumulates. All three catalytic reactions are thought to be important in the progression of cardiac fibrosis (Neff & Bradshaw, 2021). Cross‐linked collagens show collagenase resistance compared to the non‐cross‐linked type (Vater et al., 1979). In the lysyl oxidase pathway, collagen cross‐linking is initiated by the oxidation of lysine and hydroxylysine residues of collagen; the resulting allysine (α‐aminoadipic acid‐δ‐semialdehyde) and hydroxy‐allysine (hydroxy‐α‐aminoadipic acid‐δ‐semialdehyde) then form immature cross‐linking. Ultimately, further modified collagens are condensed into mature di‐ to tetra‐valent cross‐linked, degradation‐resistant structures (Eyre et al., 1984; Piersma & Bank, 2019). Transglutaminases catalyze the formation of ε (γ‐glutamyl)‐lysine cross‐link between lysine and glutamine residues in transmembrane proteins (integrins, syndecans, platelet‐derived growth factor receptor, and low‐density lipoprotein receptor‐related protein) and other transmembrane proteins or ECMs (Aeschlimann & Thomazy, 2000). The specific lysine and glutamine residues in collagens modified by transglutaminase are unclear (Neff & Bradshaw, 2021). Advanced glycation end products glycate lysine residues in fibrous collagen, competing with lysyl oxidase for some lysine residues (Saito & Marumo, 2013). In addition, mice lacking the genes for fibromodulin (*fmod*), periostin (*postn*), thrombospondin1 (*thbs1*), or secreted protein acidic and rich in cysteine (*sparc*) show abnormal collagen content, suggesting that these molecules are involved in the regulation of collagen cross‐linking (Andenæs et al., 2018; Schellings et al., 2009; Shimazaki et al., 2008; Xia et al., 2011). In mice lacking genes involved in ECM cross‐linking or inhibiting these catalytic activities, myocardial stiffness is reduced (Neff & Bradshaw, 2021). Matrix metalloproteinases (MMPs) and cysteine protease cathepsins are responsible for degrading the ECM and regulating total collagen content. The enzymatic activities of MMPs are inhibited by tissue inhibitors of MMPs (TIMPs) (Nagase et al., 2006). The human myocardium expresses MMP1, MMP2, MMP3, MMP9, MMP13, and MMP14, which degrade collagen and other substrates (Spinale, 2002). Fibrotic collagen is the dominant cross‐linking of hydroxy‐allysine type by lysyl hydroxylase 2 (Piersma & Bank, 2019; van der Slot et al., 2005). Cathepsin K, exhibiting a cardioprotective effect, more efficiently degrades strongly cross‐linked collagen by hydroxy‐allysine compared with MMPs (Guo et al., 2018; Kafienah et al., 1998). In addition, the differences in ECM‐based cardiac stiffness are indicated to be related to the potential of heart regeneration (Hortells et al., 2019). Mammalian hearts form permanent scars after myocardial infarction. In contrast, the post‐injury scar in the heart in zebrafish disappears, and the heart regenerates (Poss et al., 2002). As the progression of the degradation‐resistant collagen is suppressed in zebrafish hearts 1 month after heart cryoinjury, the collagen cross‐linking process after myocardial infarction may differ between mammals and zebrafish (Akam et al., 2023).

Recently, we reported that the ventricles of the compact layer in cherry salmon at 29–30 months post‐fertilization contain more collagen fibers than those in masu salmon (*p* = 0.0003) (Usui et al., 2022). The non‐normalized EDPVR curve for masu salmon is drawn on the left compared with that for cherry salmon (Figure 1b). In simpler terms, the end‐diastolic pressure of masu salmon is higher than that of cherry salmon at the same ventricular volume. However, it is important to note that the ventricle of cherry salmon is larger and approximately 18 times heavier than that of masu salmon (Table 1). By considering the end‐diastolic pressure of both fish species and incorporating normalized ventricular volumes using their ventricular weights following Equations 1 and 2, their EDPVR curve positions and relative ventricular stiffness indices were reversed (Figure 1c and Table 1). Given the similarity in the expression patterns of Titin in the hearts of both fish, these findings imply that the ventricle of cherry salmon becomes stiffer than that of masu salmon, possibly due to an increase in the collagen content of the compact layer (Usui et al., 2022).

The main collagen‐producing cells in mammal and fish hearts are myofibroblasts, which differentiate from cardiac fibroblasts originating from endocardial, epicardial, and neural crest cells (Hu et al., 2022; Shyu, 2017). Cardiac fibroblasts contain an extensive sarcoplasmic reticulum, secrete certain ECMs, such as thrombospondin, osteopontin, and periostin, and express α‐smooth muscle actin (Rienks et al., 2014). In mammals, cardiac fibroblasts respond to cardiac hypertrophy, myocardial infarction, and changes in ECM components. These cells then differentiate into myofibroblasts via a process mediated by transforming growth factor‐β (TGF‐β) signal through primary cilia stimulation, Wnt signal, the Hippo pathway, inflammatory cytokines (interacting with IL11R and IL17R), DAMPs, lysophosphatidic acid, GRK2, and NFAT nuclear transfer (Davis et al., 2012; He et al., 2017; Khalil et al., 2019; Parichatikanond et al., 2020; Raake et al., 2008; Schafer et al., 2017; Shyu, 2017; Turner, 2016; van Putten et al., 2016; Villalobos et al., 2019; Xiang et al., 2017; Zhang et al., 2018; Zhou et al., 2015). In mammals, myofibroblasts are believed to contribute to heart disease by increasing myocardial stiffness (Darby et al., 2014; Tomasek et al., 2002). Similarly, fibroblasts in zebrafish hearts differentiate into myofibroblasts upon heart injury, leading to the formation of a transient collagen‐rich scar (Hu et al., 2022; Poss et al., 2002).

Rainbow trout and red‐eared slider turtles exhibit heightened collagen accumulation in their ventricles, resulting in increasing ventricular stiffness when exposed to water temperatures in the range of 4–5°C (Keen et al., 2015; Keen et al., 2016). In that case, their EDPVR curve shifts to the left and becomes steeper than that of the control group (Figure 1a). Moreover, the rainbow trout displays an increase in myocardial mass of the spongy layer under such conditions (Keen et al., 2015; Klaiman et al., 2011). Although most animals suppress cardiac contractile functions at low temperatures by reducing Ca^2+^ sensitivity, fish that have adapted to such environments maintain their cardiac functions (Churcott et al., 1994; Harrison & Bers, 1990; Klaiman et al., 2014; Liu et al., 1990; Liu et al., 1993; Shiels et al., 2000; Stephenson & Williams, 1985). Cold conditions are thought to induce cardiac remodeling driven by hemodynamic load, characterized by high blood viscosity, consequently increasing ventricular stroke volume in certain fish species (Clark & Rodnick, 1999; Graham & Farrell, 1989; Keen et al., 2015; Keen et al., 2016; Klaiman et al., 2011). The increased cardiac stiffness observed during cold acclimation is likely a compensatory mechanism to prevent excessive stretching of the myocardium under mechanical stress. The extension of the myocardium upregulates the gene expression of *col1a3* and *timp2* through TGF‐β signal and the MAPK pathway, leading to collagen accumulation in the heart (Johnston & Gillis, 2018; Johnston & Gillis, 2020; Keen et al., 2015). Furthermore, the expression levels of *mmp2*, *mmp9*, and *mmp13* decrease (Keen et al., 2015). These thermal cardiac phenotypes observed in most fish are seasonal and reversible (Klaiman et al., 2011; Shiels et al., 2011). The downregulation of type I collagens is mediated by miR‐29b (Johnston et al., 2019). Cold acclimation also induces an increase in lipid biosynthesis and β‐oxidation in rainbow trout (Driedzic et al., 1996; Driedzic & Gesser, 1994); however, the direct impact of ventricular stiffness on the cold‐associated metabolic changes remains largely unknown. Some recent reviews provide a more comprehensive understanding of temperature‐dependent cardiac remodeling (Johnston & Gillis, 2022; Keen et al., 2017).

### 3.3 The range of physiological sarcomere lengths

The range of physiological sarcomere lengths of normal mammalian cardiomyocytes between *Z*‐lines is 1.8–2.2 μm (Allen & Kentish, 1985). However, peak active tension occurs in the cardiomyocytes of rainbow trout at a sarcomere length of 2.6 μm (Shiels et al., 2006). Cardiomyocytes of Wistar rats and rainbow trout generate active tension with sarcomere length dependence, increasing Ca^2+^ sensitivity in the normal and above the physiological sarcomere length range. Rainbow trout cardiomyocytes have shown higher Ca^2+^ sensitivity than Wistar rat cardiomyocytes, generating greater active tension at physiological Ca^2+^ concentrations (Patrick et al., 2010). This responsiveness of sarcomere length suggests that ventricular myocardium properties of fish are more compliant than those of mammals. In addition, force‐sarcomere strain analysis showed that the isolated cardiomyocyte stiffness of Wistar rats was 2.8 times higher than that of red‐eared slider turtles (Honda et al., 2018).

The passive tension of mammalian cardiomyocytes within the physiological sarcomere lengths mainly depends on Titin. As the myocardial tissue elongates, collagen also contributes to resisting myocardium stretching; however, the collagen‐based passive tension is relatively small compared with that induced by Titin (Granzier & Irving, 1995). The Titin‐dependent passive tension is more pronounced in myocardial tissue that expresses a higher proportion of N2BA isoforms than in other myocardial tissues expressing N2B isoforms (Wu et al., 2000). The N2BA‐like isoform expressed in cardiomyocytes of the ventricles of rainbow trout accounts for approximately 38% of the total Titin expression; however, Titin‐based passive tension at sarcomere lengths beyond the mammalian working range was generated (Patrick et al., 2010). Titins in the ventricles of rainbow trout were modified with less phosphorylation, which may be responsible for the lower myocardial stiffness (Patrick et al., 2010).

## 4 LIMITATIONS AND CONCLUSION

In addition to ventricular stiffness, myocardial stiffness is another essential indicator in the overall assessment of cardiac stiffness (Burkhoff et al., 2005; Villalobos Lizardi et al., 2022). One major limitation of the study described in this review is that EDPVR analysis only assesses global chamber stiffness. The pressure–volume analysis does not evaluate the local tissue stiffness derived from the stress–strain relationship based on Young's and shear modulus. The stress–strain relationship analysis for intrinsic myocardial properties is developed in several tests for the anisotropic elastic myocardium, providing stiffness values for local axial, transverse, and shear properties in the microscopic structure of the myocardium, independent of ventricular size (Villalobos Lizardi et al., 2022). To ensure a comprehensive evaluation of cardiac stiffness, it is crucial to incorporate both stiffness analyses.

This review describes the calculation method of the relative ventricular stiffness indices using normalized EDPVR by plotting ventricular pressure against ventricular volume per ventricle weight. Index differences in Wistar rats and red‐eared slider turtles correlate with the number of amino acids constituting the spring functional region of Titin (Honda et al., 2018). In the cases of masu and cherry salmons, these indices are reflected by the ratio of collagen accumulation in the ventricular myocardium wall (Usui et al., 2022). Additionally, these indices also seem to correlate with the relationship between sarcomere length and passive tension production in both mammals and teleosts (Shiels et al., 2006). Notably, while comparing the relative ventricular stiffness indices using ventricular volume per ventricular weight provides a reasonable interpretation to some extent, there is currently insufficient theoretical evidence for the normalizing method. Therefore, it is imperative to approach the relative ventricular stiffness index derived from EDPVR data, utilizing ventricular volume per ventricular weight on the horizontal axis, with caution and suspicion at this time. To date, changes in ventricular weight, ventricular wall thickness, fibrosis, hemodynamics, and collagen gene expression have been analyzed in detail in mouse models of HFpEF induced by obesity through high‐fat and high‐sucrose diets as well as in some diabetic model animals (Croteau et al., 2020; Gueorguiev et al., 2001; Woodiwiss et al., 1996). Accordingly, to establish the ex vivo EDPVR analysis proposed as a method based on a scientific rationale, it should be examined to verify whether the relative ventricular stiffness indices accurately rank ventricular diastolic properties in these model animals.

Few studies have tried to compare cardiac stiffness among different species with hearts of varying sizes. Therefore, there is a need for investigation into ventricular stiffness in various chordates. In this context, physiological blood pressure difference is one of the parameters that should be considered. Generally, aquatic organisms, excluding cetaceans, have a lower aortic blood pressure than mammals (20–60 vs. 60–120 mmHg) (Nishiyama et al., 2022). The ex vivo EDPVR analysis in this review calculated and compared ventricular stiffness indices within the range of 0–20 mmHg of ventricular lumen pressure. This range includes pressures exceeding physiological end‐diastolic levels. For example, the (left) ventricle end‐diastolic pressure in humans is 5–12 mmHg, which is higher than the corresponding value in fish.

Lipids hold the potential to serve as determinants of cardiac stiffness. Lipid compositions in the heart have been studied in several animals, including mice, rats, dogs, ox, redfish, and Atlantic salmon, revealing differences among species (Connellan & Masters, 1965; Joensen & Grahl‐Nielsen, 2000; Martinez‐Rubio et al., 2012; Nazir et al., 1970; Tham et al., 2018; Wheeldon et al., 1965). It should be noted that these previous studies are not strictly comparable owing to the different extraction and analysis methods. Additionally, within the same species, cardiac lipid compositions vary with age, diet, exercise, and the increasing cardiac pressure overload induced by transverse aortic constriction (TAC) (Martinez‐Rubio et al., 2012; Tham et al., 2018). Increased ventricular stiffness has been observed in the hearts of animals treated for TAC (Richards et al., 2019; Torres et al., 2020). Furthermore, slight increases in sphingolipid contents of the heart have been demonstrated in mice after TAC surgery (Richards et al., 2019). However, further verifications are necessary to determine the contribution of increased sphingolipids and species differences in lipid compositions to ventricular stiffness.

This review proposes a provisional evaluation method for comparing ventricular stiffness, an indicator of ventricular diastolic properties, among chordates with varying ventricular sizes and shapes. This ex vivo EDPVR analysis provides the relative ventricular stiffness, disregarding blood loading and employing 0 mmHg as a baseline, to delineate the relationship between ventricular volume per 1 g of myocardium and ventricular pressure. However, the validation of this normalization method for ventricular stiffness is insufficient. Therefore, in the future, rigorous scientific evidence should be established by combining our proposed analysis with the multidimensional and multiscale cardiac stiffness evaluation methods (Villalobos Lizardi et al., 2022) pioneered by the great predecessors. This will contribute to a comprehensive assessment of ventricles across various species and confirm consistency. Interpreting physiological ventricular diastolic functions based on ventricular pressure–volume relationships in various animal ventricles, undergoing changes in their weights and lumen volumes as an adaptive process to physical growth and chronic mechanical loading of the heart, may yield clinically valuable approaches. Such approaches may include defining early determinants of ventricular dysfunction, predicting its risk, and preventing its progression. In addition, we believe that comparing cardiac diastolic properties in numerous species and the same species reared in different environments will provide new insights into the adaptive evolution of cardiac physiological function, contributing to advances in cardiac physiology.

## AUTHOR CONTRIBUTIONS

Y. U. drafted the manuscript. Y. U., A. H., K. H., and S. M. prepared the figures. Y. U., A. H., K. H., M. K., M. O., and S. M. edited and revised the manuscript. Y. U., A. H., K. H., M. K., M. O., and S. M. approved the final version of the manuscript.

## FUNDING INFORMATION

This work was supported, in part, by Japan Society for the Promotion of Science, Grant/Award Number JP22K15155 to Y.U., JP20K21453, JP20H04508, and JP16K01385 to A.H., JP21K19933, JP20H04521, and JP17H02092 to K.H., JP23H00556, JP17H06272, JP17H00859, JP26282127, and JP25560214 to S.M. (https://kaken.nii.ac.jp/ja/); research grant program of the Futaba foundation in 2021 from The FUTABA foundation to Y.U. (http://www.futaba‐zaidan.org); Research Grant from the Kawasaki Foundation in 2016 from Medical Science and Medical Welfare to A.H. (https://z.kawasaki‐m.ac.jp); Medical Research Grant in 2010 from Takeda Science Foundation to K.H. (https://www.takeda‐sci.or.jp); and Research Project Grant R03S005 and R05B016 to Y.U., R03B050, R01B054, and H30B041 to A.H., H30B016 and H27B10 to K.H., and R02B039 and H28B80 to S.M. from Kawasaki Medical School (https://kms.kms‐igakkai.com).

## CONFLICT OF INTEREST STATEMENT

The authors declare no conflicts of interest, financial, or otherwise.

## ETHICS STATEMENT

Not applicable.

## Data Availability

Source data for this study are openly available at: DOI: 10.1371/journal.pone.0267264 and DOI: 10.11482/KMJ‐E44(1)1.

## References

[phy216013-bib-0001] Aeschlimann, D. , & Thomazy, V. (2000). Protein crosslinking in assembly and remodelling of extracellular matrices: The role of transglutaminases. Connective Tissue Research, 41, 1–27. 10826705 10.3109/03008200009005638

[phy216013-bib-0002] Akam, E. A. , Bergemann, D. , Ridley, S. J. , To, S , Andrea, B. , Moon, B. , Ma, H. , Zhou, Y. , Aguirre, A. , Caravan, P. , Gonzalez‐Rosa, J. M. , & Sosnovik, D. E. (2023). Dynamics of collagen oxidation and cross linking in regenerating and irreversibly infarcted myocardium. bioRxiv. 10.1101/2023.07.25.549713 PMC11164919 38858347

[phy216013-bib-0003] Allen, D. G. , & Kentish, J. C. (1985). The cellular basis of the length‐tension relation in cardiac muscle. Journal of Molecular and Cellular Cardiology, 17, 821–840. 3900426 10.1016/s0022-2828(85)80097-3

[phy216013-bib-0004] Andenæs, K. , Lunde, I. G. , Mohammadzadeh, N. , Dahl, C. P. , Aronsen, J. M. , Strand, M. E. , Palmero, S. , Sjaastad, I. , Christensen, G. , Engebretsen, K. V. T. , & Tønnessen, T. (2018). The extracellular matrix proteoglycan fibromodulin is upregulated in clinical and experimental heart failure and affects cardiac remodeling. PLoS One, 13, e0201422. 30052659 10.1371/journal.pone.0201422 PMC6063439

[phy216013-bib-0005] Bang, M. L. , Centner, T. , Fornoff, F. , Geach, A. J. , Gotthardt, M. , Mcnabb, M. , Witt, C. C. , Labeit, D. , Gregorio, C. C. , Granzier, H. , & Labeit, S. (2001). The complete gene sequence of titin, expression of an unusual approximately 700‐kDa titin isoform, and its interaction with obscurin identify a novel Z‐line to I‐band linking system. Circulation Research, 89, 1065–1072. 11717165 10.1161/hh2301.100981

[phy216013-bib-0006] Bashey, R. I. , Martinez‐Hernandez, A. , & Jimenez, S. A. (1992). Isolation, characterization, and localization of cardiac collagen type VI. Associations with other extracellular matrix components. Circulation Research, 70, 1006–1017. 1568294 10.1161/01.res.70.5.1006

[phy216013-bib-0007] Borlaug, B. A. (2014). The pathophysiology of heart failure with preserved ejection fraction. Nature Reviews. Cardiology, 11, 507–515. 24958077 10.1038/nrcardio.2014.83

[phy216013-bib-0008] Burkhoff, D. , Maurer, M. S. , & Packer, M. (2003). Heart failure with a normal ejection fraction. Circulation, 107, 656–658. 12578861 10.1161/01.cir.0000053947.82595.03

[phy216013-bib-0009] Burkhoff, D. , Mirsky, I. , & Suga, H. (2005). Assessment of systolic and diastolic ventricular properties via pressure‐volume analysis: A guide for clinical, translational, and basic researchers. American Journal of Physiology. Heart and Circulatory Physiology, 289, H501–H512. 16014610 10.1152/ajpheart.00138.2005

[phy216013-bib-0010] Caporizzo, M. A. , Chen, C. Y. , & Prosser, B. L. (2019). Cardiac microtubules in health and heart disease. Experimental Biology and Medicine (Maywood, N.J.), 244, 1255–1272. 10.1177/1535370219868960 PMC6880149 31398994

[phy216013-bib-0011] Caporizzo, M. A. , & Prosser, B. L. (2022). The microtubule cytoskeleton in cardiac mechanics and heart failure. Nature Reviews. Cardiology, 19, 364–378. 35440741 10.1038/s41569-022-00692-y PMC9270871

[phy216013-bib-0012] Cazorla, O. , Freiburg, A. , Helmes, M. , Centner, T. , Mcnabb, M. , Wu, Y. , Trombitas, K. , Labeit, S. , & Granzier, H. (2000). Differential expression of cardiac titin isoforms and modulation of cellular stiffness. Circulation Research, 86, 59–67. 10625306 10.1161/01.res.86.1.59

[phy216013-bib-0013] Churcott, C. S. , Moyes, C. D. , Bressler, B. H. , Baldwin, K. M. , & Tibbits, G. F. (1994). Temperature and pH effects on Ca2+ sensitivity of cardiac myofibrils: A comparison of trout with mammals. The American Journal of Physiology, 267, R62–R70. 8048646 10.1152/ajpregu.1994.267.1.R62

[phy216013-bib-0014] Clark, R. J. , & Rodnick, K. J. (1999). Pressure and volume overloads are associated with ventricular hypertrophy in male rainbow trout. The American Journal of Physiology, 277, R938–R946. 10516230 10.1152/ajpregu.1999.277.4.R938

[phy216013-bib-0015] Connellan, J. M. , & Masters, C. J. (1965). Fatty acid components of rat‐tissue lipids. The Biochemical Journal, 94, 81–84. 14342254 10.1042/bj0940081 PMC1206409

[phy216013-bib-0016] Croteau, D. , Qin, F. , Chambers, J. M. , Kallick, E. , Luptak, I. , Panagia, M. , Pimentel, D. R. , Siwik, D. A. , & Colucci, W. S. (2020). Differential effects of sacubitril/valsartan on diastolic function in mice with obesity‐related metabolic heart disease. JACC Basic Transl Sci, 5, 916–927. 33015414 10.1016/j.jacbts.2020.07.006 PMC7524781

[phy216013-bib-0017] Darby, I. A. , Laverdet, B. , Bonte, F. , & Desmouliere, A. (2014). Fibroblasts and myofibroblasts in wound healing. Clinical, Cosmetic and Investigational Dermatology, 7, 301–311. 25395868 10.2147/CCID.S50046 PMC4226391

[phy216013-bib-0018] Davis, J. , Burr, A. R. , Davis, G. F. , Birnbaumer, L. , & Molkentin, J. D. (2012). A TRPC6‐dependent pathway for myofibroblast transdifferentiation and wound healing in vivo. Developmental Cell, 23, 705–715. 23022034 10.1016/j.devcel.2012.08.017 PMC3505601

[phy216013-bib-0019] Diamond, G. , Forrester, J. S. , Hargis, J. , Parmley, W. W. , Danzig, R. , & Swan, H. (1971). Dlastolic pressure‐volume relationship in the canine left ventricle. Circulation Research, 29, 267–275. 5093286 10.1161/01.res.29.3.267

[phy216013-bib-0020] Driedzic, W. R. , Bailey, J. R. , & Sephton, D. H. (1996). Cardiac adaptations to low temperature in non‐polar teleost fish. Journal of Experimental Zoology, 275, 186–195.

[phy216013-bib-0021] Driedzic, W. R. , & Gesser, H. (1994). Energy metabolism and contractility in ectothermic vertebrate hearts: Hypoxia, acidosis, and low temperature. Physiological Reviews, 74, 221–258. 8295934 10.1152/physrev.1994.74.1.221

[phy216013-bib-0022] Eghbali, M. , & Weber, K. T. (1990). Collagen and the myocardium: Fibrillar structure, biosynthesis and degradation in relation to hypertrophy and its regression. Molecular and Cellular Biochemistry, 96, 1–14. 2146489 10.1007/BF00228448

[phy216013-bib-0023] Esmaeili, H. , Li, C. , Fu, X. , & Jung, J. P. (2020). Engineering extracellular matrix proteins to enhance cardiac regeneration after myocardial infarction. Frontiers in Bioengineering and Biotechnology, 8, 611936. 33553118 10.3389/fbioe.2020.611936 PMC7855456

[phy216013-bib-0024] Eyre, D. R. , Paz, M. A. , & Gallop, P. M. (1984). Cross‐linking in collagen and elastin. Annual Review of Biochemistry, 53, 717–748. 10.1146/annurev.bi.53.070184.003441 6148038

[phy216013-bib-0025] Farrell, A. P. (1991). From hagfish to tuna: A perspective on cardiac function in fish. Physiological Zoology, 64, 1137–1164.

[phy216013-bib-0026] Frangogiannis, N. G. (2019). The extracellular matrix in ischemic and nonischemic heart failure. Circulation Research, 125, 117–146. 31219741 10.1161/CIRCRESAHA.119.311148 PMC6588179

[phy216013-bib-0027] Frank, O. (1895). Zur Dynamik Ddes Herzmuskels. Z Biol, 32, 370–447.

[phy216013-bib-0028] Freiburg, A. , Trombitas, K. , Hell, W. , Cazorla, O. , Fougerousse, F. , Centner, T. , Kolmerer, B. , Witt, C. , Beckmann, J. S. , Gregorio, C. C. , Granzier, H. , & Labeit, S. (2000). Series of exon‐skipping events in the elastic spring region of titin as the structural basis for myofibrillar elastic diversity. Circulation Research, 86, 1114–1121. 10850961 10.1161/01.res.86.11.1114

[phy216013-bib-0029] Gaasch, W. H. , Levine, H. J. , Quinones, M. A. , & Alexander, J. K. (1976). Left ventricular compliance: Mechanisms and clinical implications. The American Journal of Cardiology, 38, 645–653. 136186 10.1016/s0002-9149(76)80015-x

[phy216013-bib-0030] Graham, M. S. , & Farrell, A. P. (1989). The effect of temperature acclimation and adrenaline on the performance of a perfused trout heart. Physiological Zoology, 62, 38–61.

[phy216013-bib-0031] Granzier, H. L. , & Irving, T. C. (1995). Passive tension in cardiac muscle: Contribution of collagen, titin, microtubules, and intermediate filaments. Biophysical Journal, 68, 1027–1044. 7756523 10.1016/S0006-3495(95)80278-X PMC1281826

[phy216013-bib-0032] Grossman, W. , Tefadouros, M. A. , Mclaurin, L. P. , Rolett, E. L. , & Young, D. T. (1973). Quantitative assessment of left ventricular diastolic stiffness in man. Circulation, 47, 567–574. 4266321 10.1161/01.cir.47.3.567

[phy216013-bib-0033] Gueorguiev, M. , Goth, M. L. , & Korbonits, M. (2001). Leptin and puberty: A review. Pituitary, 4, 79–86. 11824512 10.1023/a:1012943029127

[phy216013-bib-0034] Guo, R. , Hua, Y. , Ren, J. , Bornfeldt, K. E. , & Nair, S. (2018). Cardiomyocyte‐specific disruption of cathepsin K protects against doxorubicin‐induced cardiotoxicity. Cell Death & Disease, 9, 692. 29880809 10.1038/s41419-018-0727-2 PMC5992138

[phy216013-bib-0035] Guo, W. , Schafer, S. , Greaser, M. L. , Radke, M. H. , Liss, M. , Govindarajan, T. , Maatz, H. , Schulz, H. , Li, S. , Parrish, A. M. , Dauksaite, V. , Vakeel, P. , Klaassen, S. , Gerull, B. , Thierfelder, L. , Regitz‐Zagrosek, V. , Hacker, T. A. , Saupe, K. W. , Dec, G. W. , … Gotthardt, M. (2012). RBM20, a gene for hereditary cardiomyopathy, regulates titin splicing. Nature Medicine, 18, 766–773. 10.1038/nm.2693 PMC3569865 22466703

[phy216013-bib-0036] Hanashima, A. , Hashimoto, K. , Ujihara, Y. , Honda, T. , Yobimoto, T. , Kodama, A. , & Mohri, S. (2017). Complete primary structure of the I‐band region of connectin at which mechanical property is modulated in zebrafish heart and skeletal muscle. Gene, 596, 19–26. 27725266 10.1016/j.gene.2016.10.010

[phy216013-bib-0037] Harrison, S. M. , & Bers, D. M. (1990). Temperature dependence of myofilament Ca sensitivity of rat, Guinea pig, and frog ventricular muscle. The American Journal of Physiology, 258, C274–C281. 2305870 10.1152/ajpcell.1990.258.2.C274

[phy216013-bib-0038] Hashimoto, K. , Kodama, A. , Sugino, M. , Yobimoto, T. , Honda, T. , Hanashima, A. , Ujihara, Y. , & Mohri, S. (2018). Nuclear connectin novex‐3 promotes proliferation of hypoxic foetal cardiomyocytes. Scientific Reports, 8, 12337. 30120340 10.1038/s41598-018-30886-9 PMC6098106

[phy216013-bib-0039] He, L. , Huang, X. , Kanisicak, O. , Li, Y. , Wang, Y. , Li, Y. , Pu, W. , Liu, Q. , Zhang, H. , Tian, X. , Zhao, H. , Liu, X. , Zhang, S. , Nie, Y. , Hu, S. , Miao, X. , Wang, Q. D. , Wang, F. , Chen, T. , … Zhou, B. (2017). Preexisting endothelial cells mediate cardiac neovascularization after injury. The Journal of Clinical Investigation, 127, 2968–2981. 28650345 10.1172/JCI93868 PMC5531398

[phy216013-bib-0040] Heeneman, S. , Cleutjens, J. P. , Faber, B. C. , Creemers, E. E. , Van Suylen, R. J. , Lutgens, E. , Cleutjens, K. B. , & Daemen, M. J. (2003). The dynamic extracellular matrix: Intervention strategies during heart failure and atherosclerosis. The Journal of Pathology, 200, 516–525. 12845619 10.1002/path.1395

[phy216013-bib-0041] Herman, D. S. , Lam, L. , Taylor, M. R. , Wang, L. , Teekakirikul, P. , Christodoulou, D. , Conner, L. , Depalma, S. R. , Mcdonough, B. , Sparks, E. , Teodorescu, D. L. , Cirino, A. L. , Banner, N. R. , Pennell, D. J. , Graw, S. , Merlo, M. , Di Lenarda, A. , Sinagra, G. , Bos, J. M. , … Seidman, C. E. (2012). Truncations of titin causing dilated cardiomyopathy. The New England Journal of Medicine, 366, 619–628. 22335739 10.1056/NEJMoa1110186 PMC3660031

[phy216013-bib-0042] Hidalgo, C. , & Granzier, H. (2013). Tuning the molecular giant titin through phosphorylation: Role in health and disease. Trends in Cardiovascular Medicine, 23, 165–171. 23295080 10.1016/j.tcm.2012.10.005 PMC3622841

[phy216013-bib-0043] Honda, T. , Ujihara, Y. , Hanashima, A. , Hashimoto, K. , Tanemoto, K. , & Mohri, S. (2018). Turtle spongious ventricles exhibit more compliant diastolic property and possess larger elastic regions of connectin in comparison to rat compact left ventricles. Kawasaki Med J, 44, 1–17.

[phy216013-bib-0044] Hortells, L. , Johansen, A. K. Z. , & Yutzey, K. E. (2019). Cardiac fibroblasts and the extracellular matrix in regenerative and nonregenerative hearts. J Cardiovasc Dev Diseases, 6, 29. 31434209 10.3390/jcdd6030029 PMC6787677

[phy216013-bib-0045] Hu, B. , Lelek, S. , Spanjaard, B. , El‐Sammak, H. , Simoes, M. G. , Mintcheva, J. , Aliee, H. , Schafer, R. , Meyer, A. M. , Theis, F. , Stainier, D. Y. R. , Panakova, D. , & Junker, J. P. (2022). Origin and function of activated fibroblast states during zebrafish heart regeneration. Nature Genetics, 54, 1227–1237. 35864193 10.1038/s41588-022-01129-5 PMC7613248

[phy216013-bib-0133] Ito, M. , Ujihara, Y. , Sugita, S. , & Nakamura, M. (2023). Mathematical modeling and experimental analysis of the relationship between ventricular size and diastolic function of trabecular ventricles. Journal of Biorheology, 37, 96–104.

[phy216013-bib-0046] Ito, M. , Ujihara, Y. , Sugita, S. , & Nakamura, M. (2021). Comparison of the histology and stiffness of ventricles in Anura of different habitats. Journal of Biological Physics, 47, 287–300. 34515919 10.1007/s10867-021-09579-4 PMC8452808

[phy216013-bib-0047] Joensen, H. , & Grahl‐Nielsen, O. (2000). Discrimination of Sebastes viviparus, Sebastes marinus and Sebastes mentella from Faroe Islands by chemometry of the fatty acid profile in heart and gill tissues and in the skull oil. Comparative Biochemistry and Physiology. Part B, Biochemistry & Molecular Biology, 126, 69–79. 10.1016/s0305-0491(00)00172-3 10825666

[phy216013-bib-0048] Johnston, E. F. , Cadonic, I. G. , Craig, P. M. , & Gillis, T. E. (2019). microRNA‐29b knocks down collagen type I production in cultured rainbow trout (Oncorhynchus mykiss) cardiac fibroblasts. The Journal of Experimental Biology, 222, jeb202788. 31439649 10.1242/jeb.202788

[phy216013-bib-0049] Johnston, E. F. , & Gillis, T. E. (2018). Transforming growth factor‐beta1 induces differentiation of rainbow trout (Oncorhynchus mykiss) cardiac fibroblasts into myofibroblasts. The Journal of Experimental Biology, 221, jeb189167. 30397172 10.1242/jeb.189167

[phy216013-bib-0050] Johnston, E. F. , & Gillis, T. E. (2020). Short‐term cyclical stretch phosphorylates p38 and ERK1/2 MAPKs in cultured fibroblasts from the hearts of rainbow trout. Oncorhynchus mykiss, 9, bio.049296. 10.1242/bio.049296 PMC6994941 31862862

[phy216013-bib-0051] Johnston, E. F. , & Gillis, T. E. (2022). Regulation of collagen deposition in the trout heart during thermal acclimation. Current Research in Physiology, 5, 99–108. 35243359 10.1016/j.crphys.2022.02.004 PMC8857596

[phy216013-bib-0052] Kafienah, W. , Brömme, D. , Buttle, D. J. , Croucher, L. J. , & Hollander, A. P. (1998). Human cathepsin K cleaves native type I and II collagens at the N‐terminal end of the triple helix. The Biochemical Journal, 331(Pt 3), 727–732. 9560298 10.1042/bj3310727 PMC1219411

[phy216013-bib-0053] Kasner, M. , Sinning, D. , Burkhoff, D. , & Tschope, C. (2015). Diastolic pressure‐volume quotient (DPVQ) as a novel echocardiographic index for estimation of LV stiffness in HFpEF. Clinical Research in Cardiology, 104, 955–963. 25956143 10.1007/s00392-015-0863-y

[phy216013-bib-0054] Keen, A. N. , Fenna, A. J. , Mcconnell, J. C. , Sherratt, M. J. , Gardner, P. , & Shiels, H. A. (2015). The dynamic nature of hypertrophic and fibrotic remodeling of the fish ventricle. Frontiers in Physiology, 6, 427. 26834645 10.3389/fphys.2015.00427 PMC4720793

[phy216013-bib-0055] Keen, A. N. , Klaiman, J. M. , Shiels, H. A. , & Gillis, T. E. (2017). Temperature‐induced cardiac remodelling in fish. The Journal of Experimental Biology, 220, 147–160. 27852752 10.1242/jeb.128496 PMC5278617

[phy216013-bib-0056] Keen, A. N. , Shiels, H. A. , & Crossley, D. A. (2016). Cardiovascular function, compliance, and connective tissue remodeling in the turtle, Trachemys scripta, following thermal acclimation. American Journal of Physiology. Regulatory, Integrative and Comparative Physiology, 311, R133–R143. 27101300 10.1152/ajpregu.00510.2015 PMC4967230

[phy216013-bib-0057] Kellermayer, D. , Kiss, B. , Tordai, H. , Olah, A. , Granzier, H. L. , Merkely, B. , Kellermayer, M. , & Radovits, T. (2021). Increased expression of N2BA titin corresponds to more compliant myofibrils in athlete's heart. International Journal of Molecular Sciences, 22, 11110. 34681770 10.3390/ijms222011110 PMC8537917

[phy216013-bib-0058] Khalil, H. , Kanisicak, O. , Vagnozzi, R. J. , Johansen, A. K. , Maliken, B. D. , Prasad, V. , Boyer, J. G. , Brody, M. J. , Schips, T. , Kilian, K. K. , Correll, R. N. , Kawasaki, K. , Nagata, K. , & Molkentin, J. D. (2019). Cell‐specific ablation of Hsp47 defines the collagen‐producing cells in the injured heart. JCI Insight, 4, e128722. 31393098 10.1172/jci.insight.128722 PMC6693833

[phy216013-bib-0059] Klaiman, J. M. , Fenna, A. J. , Shiels, H. A. , Macri, J. , & Gillis, T. E. (2011). Cardiac remodeling in fish: Strategies to maintain heart function during temperature change. PLoS One, 6, e24464. 21915331 10.1371/journal.pone.0024464 PMC3168507

[phy216013-bib-0060] Klaiman, J. M. , Pyle, W. G. , & Gillis, T. E. (2014). Cold acclimation increases cardiac myofilament function and ventricular pressure generation in trout. The Journal of Experimental Biology, 217, 4132–4140. 25278471 10.1242/jeb.109041

[phy216013-bib-0061] Klotz, S. , Hay, I. , Dickstein, M. L. , Yi, G. H. , Wang, J. , Maurer, M. S. , Kass, D. A. , & Burkhoff, D. (2006). Single‐beat estimation of end‐diastolic pressure‐volume relationship: A novel method with potential for noninvasive application. American Journal of Physiology. Heart and Circulatory Physiology, 291, H403–H412. 16428349 10.1152/ajpheart.01240.2005

[phy216013-bib-0062] Koser, F. , Loescher, C. , & Linke, W. A. (2019). Posttranslational modifications of titin from cardiac muscle: How, where, and what for? The FEBS Journal, 286, 2240–2260. 30989819 10.1111/febs.14854 PMC6850032

[phy216013-bib-0063] Kraner, J. C. , & Ogden, E. (1956). Ventricular suction in the turtle. Circulation Research, 4, 724–726. 13365083 10.1161/01.res.4.6.724

[phy216013-bib-0064] Lahmers, S. , Wu, Y. , Call, D. R. , Labeit, S. , & Granzier, H. (2004). Developmental control of titin isoform expression and passive stiffness in fetal and neonatal myocardium. Circulation Research, 94, 505–513. 14707027 10.1161/01.RES.0000115522.52554.86

[phy216013-bib-0065] Linke, W. A. , Popov, V. I. , & Pollack, G. H. (1994). Passive and active tension in single cardiac myofibrils. Biophysical Journal, 67, 782–792. 7948691 10.1016/S0006-3495(94)80538-7 PMC1225421

[phy216013-bib-0066] Liu, B. , Wang, L. C. , & Belke, D. D. (1993). Effects of temperature and pH on cardiac myofilament Ca2+ sensitivity in rat and ground squirrel. The American Journal of Physiology, 264, R104–R108. 8430869 10.1152/ajpregu.1993.264.1.R104

[phy216013-bib-0067] Liu, B. , Wohlfart, B. , & Johansson, B. W. (1990). Effects of low temperature on contraction in papillary muscles from rabbit, rat, and hedgehog. Cryobiology, 27, 539–546. 2249456 10.1016/0011-2240(90)90041-2

[phy216013-bib-0068] Locker, R. H. , & Wild, D. J. (1986). A comparative study of high molecular weight proteins in various types of muscle across the animal kingdom. Journal of Biochemistry, 99, 1473–1484. 3754864 10.1093/oxfordjournals.jbchem.a135617

[phy216013-bib-0069] Martinez‐Rubio, L. , Morais, S. , Evensen, O. , Wadsworth, S. , Ruohonen, K. , Vecino, J. L. , Bell, J. G. , & Tocher, D. R. (2012). Functional feeds reduce heart inflammation and pathology in Atlantic salmon (Salmo salar L.) following experimental challenge with Atlantic salmon reovirus (ASRV). PLoS One, 7, e40266. 23226193 10.1371/journal.pone.0040266 PMC3511526

[phy216013-bib-0070] Maruyama, K. , Natori, R. , & Nonomura, Y. (1976). New elastic protein from muscle. Nature, 262, 58–60. 934326 10.1038/262058a0

[phy216013-bib-0071] Maurer, M. S. , Kronzon, I. , & Burkhoff, D. (2006). Ventricular pump function in heart failure with normal ejection fraction: Insights from pressure‐volume measurements. Progress in Cardiovascular Diseases, 49, 182–195. 17084178 10.1016/j.pcad.2006.08.007

[phy216013-bib-0072] Mirsky, I. (1976). Assessment of passive elastic stiffness of cardiac muscle: Mathematical concepts, physiologic and clinical considerations, directions of future research. Progress in Cardiovascular Diseases, 18, 277–308. 128035 10.1016/0033-0620(76)90023-2

[phy216013-bib-0073] Mirsky, I. (1984). Assessment of diastolic function: Suggested methods and future considerations. Circulation, 69, 836–841. 6697466 10.1161/01.cir.69.4.836

[phy216013-bib-0074] Mirsky, I. , & Pasipoularides, A. (1980). Elastic properties of normal and hypertrophied cardiac muscle. Federation Proceedings, 39, 156–161. 6444389

[phy216013-bib-0075] Mirsky, I. , & Pasipoularides, A. (1990). Clinical assessment of diastolic function. Progress in Cardiovascular Diseases, 32, 291–318. 2405455 10.1016/0033-0620(90)90018-w

[phy216013-bib-0076] Mirsky, I. , Tajimi, T. , & Peterson, K. L. (1987). The development of the entire end‐systolic pressure‐volume and ejection fraction‐afterload relations: A new concept of systolic myocardial stiffness. Circulation, 76, 343–356. 3608122 10.1161/01.cir.76.2.343

[phy216013-bib-0077] Nagase, H. , Visse, R. , & Murphy, G. (2006). Structure and function of matrix metalloproteinases and TIMPs. Cardiovascular Research, 69, 562–573. 16405877 10.1016/j.cardiores.2005.12.002

[phy216013-bib-0078] Nauroy, P. , Hughes, S. , Naba, A. , & Ruggiero, F. (2018). The in‐silico zebrafish matrisome: A new tool to study extracellular matrix gene and protein functions. Matrix Biology, 65, 5–13. 28739138 10.1016/j.matbio.2017.07.001

[phy216013-bib-0079] Nazir, D. J. , Alcaraz, A. P. , & Nair, P. P. (1970). Fatty acid composition of lipid classes from canine heart muscle subcellular fractions. Journal of Medicine, 1, 327–344. 5286502

[phy216013-bib-0080] Neff, L. S. , & Bradshaw, A. D. (2021). Cross your heart? Collagen cross‐links in cardiac health and disease. Cellular Signalling, 79, 109889. 33347984 10.1016/j.cellsig.2020.109889 PMC8830414

[phy216013-bib-0081] Nikolov, A. , & Popovski, N. (2022). Extracellular matrix in heart disease: Focus on circulating collagen type I and III derived peptides as biomarkers of myocardial fibrosis and their potential in the prognosis of heart failure: A concise review. Metabolites, 12, 297. 35448484 10.3390/metabo12040297 PMC9025448

[phy216013-bib-0082] Nishiyama, A. , Kitada, K. , & Suzuki, M. (2022). Blood pressure adaptation in vertebrates: Comparative biology. Kidney International, 102, 242–247. 35671910 10.1016/j.kint.2022.03.032

[phy216013-bib-0083] Parichatikanond, W. , Luangmonkong, T. , Mangmool, S. , & Kurose, H. (2020). Therapeutic targets for the treatment of cardiac fibrosis and cancer: Focusing on TGF‐beta signaling. Frontiers in Cardiovascular Medicine, 7, 34. 32211422 10.3389/fcvm.2020.00034 PMC7075814

[phy216013-bib-0084] Patrick, S. M. , Hoskins, A. C. , Kentish, J. C. , White, E. , Shiels, H. A. , & Cazorla, O. (2010). Enhanced length‐dependent Ca2+ activation in fish cardiomyocytes permits a large operating range of sarcomere lengths. Journal of Molecular and Cellular Cardiology, 48, 917–924. 20170661 10.1016/j.yjmcc.2010.02.008

[phy216013-bib-0085] Patterson, S. W. , & Starling, E. H. (1914). On the mechanical factors which determine the output of the ventricles. The Journal of Physiology, 48, 357–379. 16993262 10.1113/jphysiol.1914.sp001669 PMC1420422

[phy216013-bib-0086] Peterson, K. L. , Tsuji, J. , Johnson, A. , Didonna, J. , & Lewinter, M. (1978). Diastolic left ventricular pressure‐volume and stress‐strain relations in patients with valvular aortic stenosis and left ventricular hypertrophy. Circulation, 58, 77–89. 148335 10.1161/01.cir.58.1.77

[phy216013-bib-0087] Piersma, B. , & Bank, B. A. (2019). Collagen cross‐linking mediated by lysyl hydroxylase 2: An enzymatic battlefield to combat fibrosis. Essays in Biochemistry, 63, 377–387. 31324706 10.1042/EBC20180051

[phy216013-bib-0088] Poss, K. D. , Wilson, L. G. , & Keating, M. T. (2002). Heart regeneration in zebrafish. Science, 298, 2188–2190. 12481136 10.1126/science.1077857

[phy216013-bib-0089] Raake, P. W. , Vinge, L. E. , Gao, E. , Boucher, M. , Rengo, G. , Chen, X. , Degeorge, B. R. , Matkovich, S. , Houser, S. R. , Most, P. , Eckhart, A. D. , Dorn, G. W. , & Koch, W. J. (2008). G protein‐coupled receptor kinase 2 ablation in cardiac myocytes before or after myocardial infarction prevents heart failure. Circulation Research, 103, 413–422. 18635825 10.1161/CIRCRESAHA.107.168336 PMC2679955

[phy216013-bib-0090] Ricard‐Blum, S. (2011). The collagen family. Cold Spring Harbor Perspectives in Biology, 3, a004978. 21421911 10.1101/cshperspect.a004978 PMC3003457

[phy216013-bib-0091] Richards, D. A. , Aronovitz, M. J. , Calamaras, T. D. , Tam, K. , Martin, G. L. , Liu, P. , Bowditch, H. K. , Zhang, P. , Huggins, G. S. , & Blanton, R. M. (2019). Distinct phenotypes induced by three degrees of transverse aortic constriction in mice. Scientific Reports, 9, 5844. 30971724 10.1038/s41598-019-42209-7 PMC6458135

[phy216013-bib-0092] Rienks, M. , Papageorgiou, A.‐P. , Frangogiannis, N. G. , & Heymans, S. (2014). Myocardial extracellular matrix. Circulation Research, 114, 872–888. 24577967 10.1161/CIRCRESAHA.114.302533

[phy216013-bib-0093] Saito, M. , & Marumo, K. (2013). Bone quality in diabetes. Frontiers in Endocrinology, 4, 72. 23785354 10.3389/fendo.2013.00072 PMC3682213

[phy216013-bib-0094] Sarohi, V. , Srivastava, S. , & Basak, T. (2022). Comprehensive mapping and dynamics of site‐specific prolyl‐hydroxylation, lysyl‐hydroxylation and lysyl O‐glycosylation of collagens deposited in ECM during zebrafish heart regeneration. Frontiers in Molecular Biosciences, 9, 892763. 35782869 10.3389/fmolb.2022.892763 PMC9245515

[phy216013-bib-0095] Schafer, S. , Viswanathan, S. , Widjaja, A. A. , Lim, W. W. , Moreno‐Moral, A. , Delaughter, D. M. , Ng, B. , Patone, G. , Chow, K. , Khin, E. , Tan, J. , Chothani, S. P. , Ye, L. , Rackham, O. J. L. , Ko, N. S. J. , Sahib, N. E. , Pua, C. J. , Zhen, N. T. G. , Xie, C. , … Cook, S. A. (2017). IL‐11 is a crucial determinant of cardiovascular fibrosis. Nature, 552, 110–115. 29160304 10.1038/nature24676 PMC5807082

[phy216013-bib-0096] Schellings, M. W. , Vanhoutte, D. , Swinnen, M. , Cleutjens, J. P. , Debets, J. , Van Leeuwen, R. E. , D'hooge, J. , Van De Werf, F. , Carmeliet, P. , Pinto, Y. M. , Sage, E. H. , & Heymans, S. (2009). Absence of SPARC results in increased cardiac rupture and dysfunction after acute myocardial infarction. The Journal of Experimental Medicine, 206, 113–123. 19103879 10.1084/jem.20081244 PMC2626676

[phy216013-bib-0097] Schwarzl, M. , Ojeda, F. , Zeller, T. , Seiffert, M. , Becher, P. M. , Munzel, T. , Wild, P. S. , Blettner, M. , Lackner, K. J. , Pfeiffer, N. , Beutel, M. E. , Blankenberg, S. , & Westermann, D. (2016). Risk factors for heart failure are associated with alterations of the LV end‐diastolic pressure‐volume relationship in non‐heart failure individuals: Data from a large‐scale, population‐based cohort. European Heart Journal, 37, 1807–1814. 27055814 10.1093/eurheartj/ehw120

[phy216013-bib-0098] Shah, S. J. , Borlaug, B. A. , Kitzman, D. W. , Mcculloch, A. D. , Blaxall, B. C. , Agarwal, R. , Chirinos, J. A. , Collins, S. , Deo, R. C. , Gladwin, M. T. , Granzier, H. , Hummel, S. L. , Kass, D. A. , Redfield, M. M. , Sam, F. , Wang, T. J. , Desvigne‐Nickens, P. , & Adhikari, B. B. (2020). Research priorities for heart failure with preserved ejection fraction: National heart, lung, and blood institute working group summary. Circulation, 141, 1001–1026. 32202936 10.1161/CIRCULATIONAHA.119.041886 PMC7101072

[phy216013-bib-0099] Shiels, H. A. , Calaghan, S. C. , & White, E. (2006). The cellular basis for enhanced volume‐modulated cardiac output in fish hearts. The Journal of General Physiology, 128, 37–44. 16769795 10.1085/jgp.200609543 PMC2151555

[phy216013-bib-0100] Shiels, H. A. , Di Maio, A. , Thompson, S. , & Block, B. A. (2011). Warm fish with cold hearts: Thermal plasticity of excitation‐contraction coupling in bluefin tuna. Proceedings of the Biological Sciences, 278, 18–27. 10.1098/rspb.2010.1274 PMC2992732 20667881

[phy216013-bib-0101] Shiels, H. A. , Vornanen, M. , & Farrell, A. P. (2000). Temperature‐dependence of L‐type Ca^2+^ channel current in atrial myocytes from rainbow trout. The Journal of Experimental Biology, 203, 2771–2780. 10952877 10.1242/jeb.203.18.2771

[phy216013-bib-0102] Shimazaki, M. , Nakamura, K. , Kii, I. , Kashima, T. , Amizuka, N. , Li, M. , Saito, M. , Fukuda, K. , Nishiyama, T. , Kitajima, S. , Saga, Y. , Fukayama, M. , Sata, M. , & Kudo, A. (2008). Periostin is essential for cardiac healing after acute myocardial infarction. The Journal of Experimental Medicine, 205, 295–303. 18208976 10.1084/jem.20071297 PMC2271007

[phy216013-bib-0103] Shyu, K. G. (2017). The role of endoglin in myocardial fibrosis. Acta Cardiol Sin, 33, 461–467. 28959097 10.6515/ACS20170221B PMC5611341

[phy216013-bib-0104] Spinale, F. G. (2002). Matrix metalloproteinases: Regulation and dysregulation in the failing heart. Circulation Research, 90, 520–530. 11909815 10.1161/01.res.0000013290.12884.a3

[phy216013-bib-0105] Stephenson, D. G. , & Williams, D. A. (1985). Temperature‐dependent calcium sensitivity changes in skinned muscle fibres of rat and toad. The Journal of Physiology, 360, 1–12. 3921690 10.1113/jphysiol.1985.sp015600 PMC1193444

[phy216013-bib-0106] Suga, H. (1969). Time course of left ventricular pressure‐volume relationship under various enddiastolic volume. Japanese Heart Journal, 10, 509–515. 5308142 10.1536/ihj.10.509

[phy216013-bib-0107] Suga, H. (1979). Total mechanical energy of a ventricle model and cardiac oxygen consumption. The American Journal of Physiology, 236, H498–H505. 426086 10.1152/ajpheart.1979.236.3.H498

[phy216013-bib-0108] Suga, H. (1990). Ventricular energetics. Physiological Reviews, 70, 247–277. 2181496 10.1152/physrev.1990.70.2.247

[phy216013-bib-0109] Suga, H. , Hisano, R. , Goto, Y. , & Yamada, O. (1984). Normalization of end‐systolic pressure‐volume relation and Emax of different sized hearts. Japanese Circulation Journal, 48, 136–143. 6700110 10.1253/jcj.48.136

[phy216013-bib-0110] Takaoka, H. , Esposito, G. , Mao, L. , Suga, H. , & Rockman, H. A. (2002). Heart size‐independent analysis of myocardial function in murine pressure overload hypertrophy. American Journal of Physiology. Heart and Circulatory Physiology, 282, H2190–H2197. 12003828 10.1152/ajpheart.00759.2001

[phy216013-bib-0111] Tham, Y. K. , Bernardo, B. C. , Huynh, K. , Ooi, J. Y. Y. , Gao, X. M. , Kiriazis, H. , Giles, C. , Meikle, P. J. , & Mcmullen, J. R. (2018). Lipidomic profiles of the heart and circulation in response to exercise versus cardiac pathology: A resource of potential biomarkers and drug targets. Cell Reports, 24, 2757–2772. 30184508 10.1016/j.celrep.2018.08.017

[phy216013-bib-0112] Tomasek, J. J. , Gabbiani, G. , Hinz, B. , Chaponnier, C. , & Brown, R. A. (2002). Myofibroblasts and mechano‐regulation of connective tissue remodelling. Nature Reviews. Molecular Cell Biology, 3, 349–363. 11988769 10.1038/nrm809

[phy216013-bib-0113] Torres, W. M. , Barlow, S. C. , Moore, A. , Freeburg, L. A. , Hoenes, A. , Doviak, H. , Zile, M. R. , Shazly, T. , & Spinale, F. G. (2020). Changes in myocardial microstructure and mechanics with progressive left ventricular pressure overload. JACC Basic Transl Sci, 5, 463–480. 32478208 10.1016/j.jacbts.2020.02.007 PMC7251228

[phy216013-bib-0114] Trombitas, K. , Wu, Y. , Labeit, D. , Labeit, S. , & Granzier, H. (2001). Cardiac titin isoforms are coexpressed in the half‐sarcomere and extend independently. American Journal of Physiology. Heart and Circulatory Physiology, 281, H1793–H1799. 11557573 10.1152/ajpheart.2001.281.4.H1793

[phy216013-bib-0115] Turner, N. A. (2016). Inflammatory and fibrotic responses of cardiac fibroblasts to myocardial damage associated molecular patterns (DAMPs). Journal of Molecular and Cellular Cardiology, 94, 189–200. 26542796 10.1016/j.yjmcc.2015.11.002

[phy216013-bib-0116] Usui, Y. , Kimoto, M. , Hanashima, A. , Hashimoto, K. , & Mohri, S. (2022). Cardiac hemodynamics and ventricular stiffness of sea‐run cherry salmon (Oncorhynchus masou masou) differ critically from those of landlocked masu salmon. PLoS One, 17, e0267264. 36331913 10.1371/journal.pone.0267264 PMC9635730

[phy216013-bib-0117] Van Der Slot, A. J. , Van Dura, E. A. , De Wit, E. C. , De Groot, J. , Huizinga, T. W. , Bank, R. A , & Zuurmond, A. M. (2005). Elevated formation of pyridinoline cross‐links by profibrotic cytokines is associated with enhanced lysyl hydroxylase 2b levels. Biochimica et Biophysica Acta, 1741, 95–102. 15955452 10.1016/j.bbadis.2004.09.009

[phy216013-bib-0118] Van Putten, S. , Shafieyan, Y. , & Hinz, B. (2016). Mechanical control of cardiac myofibroblasts. Journal of Molecular and Cellular Cardiology, 93, 133–142. 26620422 10.1016/j.yjmcc.2015.11.025

[phy216013-bib-0119] Vater, C. A. , Harris, E. D. , & Siegel, R. C. (1979). Native cross‐links in collagen fibrils induce resistance to human synovial collagenase. The Biochemical Journal, 181, 639–645. 42386 10.1042/bj1810639 PMC1161203

[phy216013-bib-0120] Villalobos, E. , Criollo, A. , Schiattarella, G. G. , Altamirano, F. , French, K. M. , May, H. I. , Jiang, N. , Nguyen, N. U. N. , Romero, D. , Roa, J. C. , Garcia, L. , Diaz‐Araya, G. , Morselli, E. , Ferdous, A. , Conway, S. J. , Sadek, H. A. , Gillette, T. G. , Lavandero, S. , & Hill, J. A. (2019). Fibroblast primary cilia are required for cardiac fibrosis. Circulation, 139, 2342–2357. 30818997 10.1161/CIRCULATIONAHA.117.028752 PMC6517085

[phy216013-bib-0121] Villalobos Lizardi, J. C. , Baranger, J. , Nguyen, M. B. , Asnacios, A. , Malik, A. , Lumens, J. , Mertens, L. , Friedberg, M. K. , Simmons, C. A. , Pernot, M. , & Villemain, O. (2022). A guide for assessment of myocardial stiffness in health and disease. Nature Cardiovascular Research, 1, 8–22. 10.1038/s44161-021-00007-3 39196108

[phy216013-bib-0122] Wang, K. , Mcclure, J. , & Tu, A. (1979). Titin: Major myofibrillar components of striated muscle. Proceedings of the National Academy of Sciences of the United States of America, 76, 3698–3702. 291034 10.1073/pnas.76.8.3698 PMC383900

[phy216013-bib-0123] Warburton, S. J. , & Fritsche, R. (2000). Blood pressure control in a larval amphibian, Xenopus laevis. The Journal of Experimental Biology, 203, 2047–2052. 10851121 10.1242/jeb.203.13.2047

[phy216013-bib-0124] Wheeldon, L. W. , Schumert, Z. , & Turner, D. A. (1965). Lipid composition of heart muscle homogenate. Journal of Lipid Research, 6, 481–489. 4286162

[phy216013-bib-0125] Wisneski, J. A. , & Bristow, J. D. (1978). Left ventricular stiffness. Annual Review of Medicine, 29, 475–483. 10.1146/annurev.me.29.020178.002355 348043

[phy216013-bib-0126] Woodiwiss, A. J. , Kalk, W. J. , & Norton, G. R. (1996). Habitual exercise attenuates myocardial stiffness in diabetes mellitus in rats. The American Journal of Physiology, 271, H2126–H2133. 8945933 10.1152/ajpheart.1996.271.5.H2126

[phy216013-bib-0127] Wu, Y. , Cazorla, O. , Labeit, D. , Labeit, S. , & Granzier, H. (2000). Changes in titin and collagen underlie diastolic stiffness diversity of cardiac muscle. Journal of Molecular and Cellular Cardiology, 32, 2151–2162. 11112991 10.1006/jmcc.2000.1281

[phy216013-bib-0128] Wu, Y. , Tobias, A. H. , Bell, K. , Barry, W. , Helmes, M. , Trombitas, K. , Tucker, R. , Campbell, K. B. , & Granzier, H. L. (2004). Cellular and molecular mechanisms of systolic and diastolic dysfunction in an avian model of dilated cardiomyopathy. Journal of Molecular and Cellular Cardiology, 37, 111–119. 15242741 10.1016/j.yjmcc.2004.04.010

[phy216013-bib-0129] Xia, Y. , Dobaczewski, M. , Gonzalez‐Quesada, C. , Chen, W. , Biernacka, A. , Li, N. , Lee, D. W. , & Frangogiannis, N. G. (2011). Endogenous thrombospondin 1 protects the pressure‐overloaded myocardium by modulating fibroblast phenotype and matrix metabolism. Hypertension, 58, 902–911. 21947471 10.1161/HYPERTENSIONAHA.111.175323 PMC3199343

[phy216013-bib-0130] Xiang, F. L. , Fang, M. , & Yutzey, K. E. (2017). Loss of beta‐catenin in resident cardiac fibroblasts attenuates fibrosis induced by pressure overload in mice. Nature Communications, 8, 712. 10.1038/s41467-017-00840-w PMC5620049 28959037

[phy216013-bib-0131] Zhang, Y. , Zhang, Y. Y. , Li, T. T. , Wang, J. , Jiang, Y. , Zhao, Y. , Jin, X. X. , Xue, G. L. , Yang, Y. , Zhang, X. F. , Sun, Y. Y. , Zhang, Z. R. , Gao, X. , Du, Z. M. , Lu, Y. J. , Yang, B. F. , & Pan, Z. W. (2018). Ablation of interleukin‐17 alleviated cardiac interstitial fibrosis and improved cardiac function via inhibiting long non‐coding RNA‐AK081284 in diabetic mice. Journal of Molecular and Cellular Cardiology, 115, 64–72. 29305939 10.1016/j.yjmcc.2018.01.001

[phy216013-bib-0132] Zhou, Q. , Li, L. , Zhao, B. , & Guan, K. L. (2015). The hippo pathway in heart development, regeneration, and diseases. Circulation Research, 116, 1431–1447. 25858067 10.1161/CIRCRESAHA.116.303311 PMC4394208

